# Systematic Review of Morphometric Analysis of Anterior Cerebral Artery (ACA) Emphasizing on Its Clinical Implications

**DOI:** 10.7759/cureus.37744

**Published:** 2023-04-18

**Authors:** Suyashi Sharma, Hare Krishna, Shilpi G Dixit, Ashish K Nayyar, Pushpinder Khera, Surajit Ghatak

**Affiliations:** 1 Anatomy, All India Institute of Medical Sciences, Jodhpur, Jodhpur, IND; 2 Diagnostic and Interventional Radiology, All India Institute of Medical Sciences, Jodhpur, Jodhpur, IND

**Keywords:** angiographic, cadaveric, radiological, digital subtraction angiography, length, diameter, morphometry, anterior cerebral artery

## Abstract

Thorough data of morphometric measurements of arteries forming Circle of Willis (CW) is crucial for radiological and neurosurgical interventions. This systematic review has been conducted with the objective to find an effective range of length and diameter of anterior cerebral artery (ACA) and to observe whether there is any change in the length and diameter of ACA depending on age or sex. Articles based on length and diameter of ACA via any mode of study like cadaveric or radiological were considered in this systematic review. A comprehensive literature search using databases Cochrane Library, PubMed, and Scopus for relevant articles was done. Research papers which answered the focused questions were selected for data analysis. It was observed that the range of length and diameter of ACA were 8.1 mm-21 mm and 0.5 Å-3.4 mm, respectively. In majority of the studies, length and diameter of ACA were more in the younger age group (>40 years); and the length of ACA was more in females whereas the diameter of ACA was more in males. These data will be applicable for better construction and decipherment of angiographic images. This will help in the proper and guided treatment of intracranial pathologies.

## Introduction and background

The supply of nutrients to the brain remains unceased for the brain to be alive. Circle of Willis (CW) is the safest tool for the brain. If there is any unilateral blockage in the arterial supply to the brain, it will be compensated by supply via CW [1]. The knowledge of vessels of the brain is substantial for the neurosurgeons operating infarction of brain tissue due to stenosis or embolism of cerebral arteries leading to blockage. A study on anterior cerebral artery (ACA) is important because occlusion can also occur in this artery (3% incidence) and it can hamper blood supply of sufficient area of brain (medial part of frontal and parietal lobes) [2]. Moreover, hypoplasia of ACA has been a critical condition for the neurosurgeons to manage [3].

The ACA supplies blood to the medial cerebral hemisphere (most midline area of frontal lobe and superior medial parietal lobes of brain) which is responsible for perception and understanding. Blockage of this artery can lead to disconnection of non-dominant hemisphere from centers concerned with the organization of speech and skilled motor activity in the dominant hemisphere resulting in agraphia and apraxia [4].

Conjunction between the internal carotid and vertebral artery at the base of brain is known as the CW. In front of CW lies ACA which is the terminal branch of internal carotid artery (ICA). The ACA of both sides are connected to each other by anterior communicating artery (ACoA). Posterior cerebral arteries (PCA) lie behind in CW. PCA is the terminal branch of basilar artery. PCA of a side is connected to ICA of the same side by the posterior communicating artery (PCoA) [1]. Most of the branches of CW formed from ICA, develop from the third pharyngeal arch artery. ICA ends with a rostral and a caudal division. At 6th-7th week of embryological life, the medial branch of the rostral segment is the most primitive form of ACA complex in human beings [5].

The present study aims to ascertain various studies concerning morphometric measurements of ACA. Study of ACA is crucial during surgeries of the anterior part of CW. ACA is the most common vessel for the development of intracranial aneurysms [6]. The capability of ancillary functioning of CW depends on the size of the arteries forming CW [7].

We did not find any systematic review on the morphometric evaluation of ACA. This systematic review included research articles that had evaluated the length and/or diameter of ACA via cadaveric or angiographic study like digital subtraction angiography (DSA), computed tomography angiography (CTA), and magnetic resonance angiography (MRA). The data from various studies are pooled based on age and gender. Hence, a systematic review was conducted to find an effective range of length and diameter of ACA in order to help the neurosurgeons while dealing with complexities like aneurysm, arterial dissection, etc. Morphometry of the artery helps in choosing the correct size of catheters for endovascular interventions [4].

## Review

Methods

This systematic review was conducted with the objective to find an effective range of length and diameter of ACA and to observe whether there is any change in the length and diameter of ACA depending on age or sex.

Review Question

Is there any dimorphism related to age and sex in morphometry of the anterior cerebral artery? This systematic review was based on articles in accordance with the above review question. “PRISMA Statement 2020” (Preferred Reporting Items for Systematic Reviews and Meta-Analysis) guidelines were used for this present study [8]. We followed PICO (Population, Intervention, Comparison and Outcome) for the review question (Table 1) [9].

**Table 1:** Eligibility criteria for inclusion of the studies. ACA, anterior cerebral artery

PICOS	Inclusion criteria	Exclusion criteria
Population	Adult of any age, gender and ethnicity	Adult having cerebrovascular malformation
Intervention	Morphometric evaluation of ACA	Morphometric evaluation of pathological segments of ACA
Comparison	No control	-
Outcomes	Length and/or diameter of ACA	Any other evaluation

All the studies included in this systematic review were observational (cross-sectional) studies. PRISMA 2020 guidelines were followed for screening and selecting the studies for this systematic review. Articles were selected after a comprehensive database search. The methodological quality of the studies was done (via ROBIS tool) [10] (Table 2). 

**Table 2:** Risk of bias summary. Review authors’ judgements about each risk of bias item for each included study. [Low risk of bias (-), High risk of bias (+), Unclear risk of bias (?)]

Study	Random sequence generation	Allocation concealment	Blinding of participants	Blinding of outcome assessment	Incomplete outcome data	Selective reporting
Karatas et al. (2015) [11]	-	?	-	-	-	-
Shatri et al. (2017) [12]	-	?	-	-	-	-
Orlandini et al. (1985) [13]	+	+	+	-	-	-
Canaz et al. (2018) [14]	-	?	?	?	-	-
Pai et al. (2005) [15]	-	-	-	-	-	-
Thenmozhi et al. (2019) [16]	-	?	?	-	-	-
Kamath S (1981) [17]	-	+	-	-	-	-
Shatri et al. (2018) [18]	-	-	-	-	+	?
Sharma et al. (2020) [19]	-	-	-	-	+	+
Murray (1964) [20]	+	+	+	+	+	+
Mandiola et al. (2007) [21]	-	-	-	-	+	+
Smita et al. (2016) [22]	-	-	-	-	+	+
Kedia et al. (2013) [23]	-	+	-	-	-	-
Gomes et al. (1986) [24]	+	-	-	-	-	-
Aggarwal et al. (2012) [25]	-	-	-	-	?	?
Karatas et al. (2015) [26]	-	-	-	?	-	-
Vohra et al. (2006) [27]	-	-	-	-	-	-
Perlmutter et al. (1976) [28]	-	-	-	+	+	?
Aggarwal et al. (2016) [29]	-	-	-	-	-	-
Hartkamp et al. (1998) [30]	-	-	-	+	-	-
Maaly et al. (2011) [31]	+	-	-	-	-	?

Literature Search and Data Extraction

We conducted a comprehensive literature search using databases Cochrane Library, PubMed, and Scopus for relevant articles. The search was limited to papers published in English from 1st January 1964 until 31st December 2022. Articles based on length and diameter of ACA were considered via any mode of study like cadaveric or radiological. The keywords used in the literature search were “Anterior cerebral artery” AND “Morphometry,” “Anterior cerebral artery” AND “Angiography”, “Anterior cerebral artery” AND “Morphometry” AND “Angiography”, “Anterior cerebral artery” AND “Morphometry” AND “Cadaver”, “Intracranial” AND “Morphometry”, “Length” AND “Anterior cerebral artery”, “Diameter” AND “Anterior cerebral artery”. Articles appearing in multiple databases were considered only once. Duplicates were sorted out by using Endnote X7 software for managing references. Title and abstracts were screened carefully and studies that were related to this study were included. Only the articles in the English language were considered. Initially, 61 articles were selected, 17 of them were found unrelated, and seven were not full-text hence excluded from the study. Finally, after exclusion of duplicates, unrelated articles and not full text articles; 21 articles were considered in this systematic review. The largest study had 513 cases while the smallest study had 10 cases. Only full texted articles were included and otherwise excluded and disagreement was also resolved through discussions of author 1 and author 2 of this systematic review. Full-text eligibility was independently analyzed by screening the title and abstract by two researchers. The final decision was taken after discussion with the other authors. All articles included in this review were checked twice by the authors. It was noticed that in the cadaveric studies measurements were taken using Vernier calipers, while the radiological studies had used the imaging technique of angiography.

 *Inclusion Criteria*

-Cross-sectional studies which had reported the length and/or diameter of ACA were included for our systematic review.

-Studies which reported morphometric evaluation of ACA in adults (>18 years of age).

Exclusion Criteria

-All the studies which were not in the English language were excluded.

-Studies involving the measurement of pathological segments of ACA (dysplasia, aneurysms, dissections) were not included.

Assessment of Quality of Study

The risk of bias was calculated by the ROBIS tool. Each study was rated “low concern,” “high concern,” or “unclear concern” by ROBIS. “Low concern” relates to the articles with clear specifications of the review question and objectives. “High concern” refers to the studies with a lack of pre-specified objectives for addressing the review question. Studies with insufficient information to make a judgement about the risk of bias lies in the category of “unclear concern” [10].

Result

**Figure 1:**
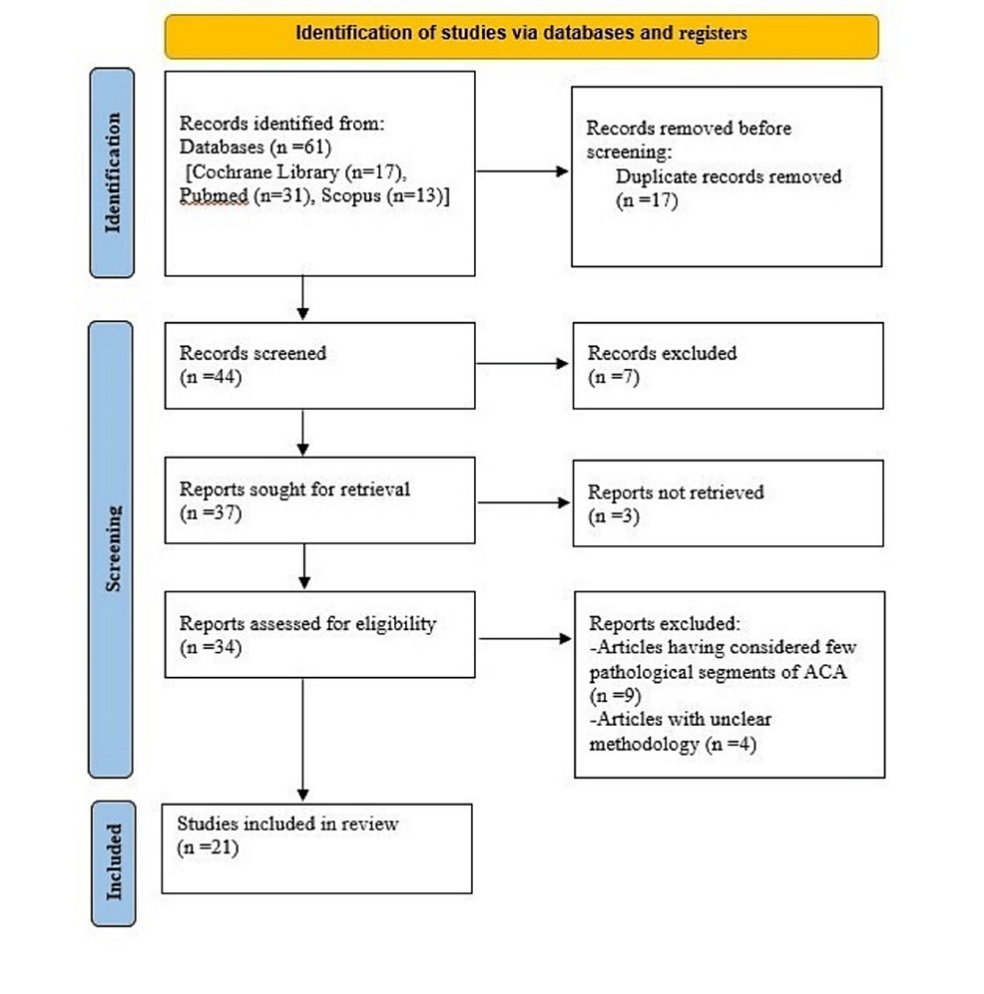
Flowchart of the article selection (PRISMA 2020 flow diagram). n, number of articles

All the studies included in this systematic review were cross-sectional studies. The review was conducted in accordance with the “PRISMA Statement 2020” [7] (Figure 1). Assessment of the quality of the studies was done via ROBIS tool [9] (Table 2). Out of the total 21 studies, nine (42.9%) were cadaveric and the remaining (57.1%) were radiological studies. Among the radiological studies all of them were done via MRA except two (one via CTA and one via DSA). Some 12 (57.1%) studies had observed both the length and diameter of ACA, while the remaining nine (42.9%) observed either length or diameter of ACA.

Length of ACA

A varying range of length was found in the included studies. After observing the results of the range of length of all the included articles, we found a minimum value of 8.1 mm and a maximum of 21 mm. Among all the included studies, minimum (8.1 mm) and maximum (21 mm) values of length of ACA were recorded in the study of Karatas et al. and Shatri et al. respectively [11-12]. Dimension of the length of ACA was similar in the studies of Orlandini et al., Canaz et al., Pai et al., Thenmozhi et al., Kamath et al., Shatri et al., and Sharma et al. having the range between 13.5 mm and 14.5 mm [13-19]. While the findings of Mu et al., Mandiola et al., Smita BS et al. and Kedia et al. had the range 12- 13 mm of the length of ACA [20,21,22,23]. Moreover results of Gomes et al. and Aggarwal N et al. found ACA length to be in the range of 15 mm; that is higher than the findings of other studies [24,25]. Karatas A et al. came up with two studies in 2015, one cadaveric and the other one via CTA [11,26]. Both his studies gave wide range of length of ACA (8 mm -20 mm), thus differing from the remaining studies.

Sex-Wise Distribution of ACA Length

One study was found that observed the gender-wise distribution of length of ACA. Sharma S et al. differed and concluded ACA length to be more in females [19].

Age-Wise Distribution of Length of ACA

Two of the included studies considered age as the criteria for observing the length of ACA. Studies of Shatri et al. and Sharma S et al. supported each other’s conclusion of length being more in the younger age group [12, 19].

Diameter of ACA

On analyzing the range of observed diameter among the included studies, it was noticed that the minimum and maximum values were 0.5Å and 3.4 mm respectively. Cadaveric studies of Karatas et al. and Vohra et al. observed both the minimum (0.05 mm) and maximum (3.4 mm) values of diameter [26,27]. The diameter of ACA was similar in the findings of Kamath et al., Sharma et al., Mandiola et al., Smita et al., Kardile et al., Gomes et al., Perlmutter et al. (2.5 mm); whereas significant difference was obtained in the values of studies by Canaz et al. and Pai et al. [14-15, 17, 19, 21-24, 28]. Canaz et al. came up with the diameter in the range of 1.5 mm while Pai et al. had much higher value (3 mm) than the other studies [14-15]. Shatri et al., Karatas et al., and Vohra et al. studies showed a wide range of diameters of ACA [12, 26-27].

Sex-Wise Distribution of ACA Diameter

Four studies evaluated the diameter of ACA in males and females. Out of these four studies, three were of the opinion of diameter being more in males. The only study of Sharma et al. stated it to be more in females [19]. Whereas, the studies of Shatri et al., Aggarwal et al., and Krabbe-Hartkamp et al. concluded that the diameter of ACA is more in males [12, 29-30].

Age-Wise Distribution of Diameter of ACA

Four of the included studies observed the diameter of ACA in young and old populations (age > 40 years). Studies of Sharma et al., Krabbe-Hartkamp et al., and Maaly et al. opined that the diameter of ACA is more in younger age group [19, 30-31]. Whereas, Shatri et al. opposed these studies as their study resulted in ACA diameter being more in the older age group [12].

Overall as such no significant difference was noticed in the pattern or range of length or diameter of ACA, depending on whether the study was cadaveric or radiological. Results of studies included in our systematic review have been mentioned in Table 3.

**Table 3:** Result of length and diameter of ACA in different studies. [N.A - Not Available (study was done either for length or diameter), R - Right side, L - Left side] SD, standard deviation; CTA, computed tomography angiography; MRA, magnetic resonance angiography

Authors	Mode of study	Sample size	Result (mean length ± SD)	Result (mean diameter ± SD)	Remarks
Kamath (1981) [17]	Cadaveric study	100	R- 14.7±3.0 mm L- 13.8±2.7 mm	R- 2.2±0.6 mm L- 2.4±0.05 mm	-
Gomes et al. (1986) [24]	Cadaveric study	30	15 mm	R- 2.3±1.0 mm L- 2.5±1.0 mm	-
Pai et al. (2005) [15]	Cadaveric study	10	L- 14.5 mm R- 14.6 mm	R- 2.8 mm L- 2.9 mm	-
Mandiola et al. (2007) [21]	Cadaveric study	36	R- 12.86±1.58 mm L- 12.62±1.96 mm	R- 2.37±0.68 mm L- 2.42±0.75 mm	-
Kedia et al. (2013) [23]	Cadaveric study	15	R- 12.09 mm L- 12 mm	R- 2.32 mm L- 2.36 mm	-
Karatas et al. (2016) [11]	Cadaveric study	100	R– 14.44±2.32 mm L- 13.72±2.12 mm	R- 1.87±0.48 mm L- 1.96±0.49 mm	-
Smita et al. (2016) [22]	Cadaveric study	50	R- 12 mm L- 13 mm	R- 2.1 mm L- 2.4 mm	-
Canaz et al. (2018) [14]	Cadaveric study	30	R- 13.56±2.25 mm L- 13.76±1.87 mm	R- 1.54±0.36 mm L- 1.84±0.35 mm	-
Murray et al. (1964) [20]	Cadaveric study	35	R- 13.2 mm L- 12.9 mm	N.A.	-
Orlandini et al. (1985) [13]	Cadaveric study	100	R- 14.1 mm L- 13.6 mm	N.A.	-
Thenmozhi et al. (2019) [16]	Cadaveric study	100	R- 14.44 mm L- 13.6 mm	N.A.	-
Perlmutter et al. (1976) [28]	Cadaveric study	50	N.A.	2.6 mm	-
Karatas et al. (2015) [26]	CTA	100	R- 15.61±2.79 mm L- 15.13±2.54 mm	R- 2.15±0.63 mm L- 2.26±0.61 mm	-
Shatri et al. (2017) [12]	MRA	133	13.96±1.4 mm Younger age group- 14.32±1.65 mm Older age group- 13.6±0.96 mm	2.09±0.27 mm Males- 2.12±0.29 mm Females- 2.04±0.23 mm Younger age group- 2.02±0.31 mm Older age group- 2.07±0.26 mm	-Length is more in younger age group -Diameter is more in older age group - Diameter is more in males
Shatri et al. (2018) [18]	MRA	513	14.1 mm	R– 2.04 mm L– 2.06 mm	-
Sharma et al. (2020) [19]	DSA	70	Males- 14.04 mm Females- 14.51 mm Younger age group- 14.64 mm Older age group- 14.34 mm	Males- 2.28 mm Females- 2.33 mm Younger age group- 2.3 mm Older age group- 2.12 mm	-Length and diameter are more in females -Length and diameter are more in younger age group
Aggarwal et al. (2012) [25]	MRA	50	R- 17.37 mm L- 15.78 mm	N.A.	-
Krabbe-Hartkamp et al. (1998) [30]	MRA	75	N.A.	Younger age group Males: R- 2.3 mm L- 2.2 mm Females: R- 2.3 mm L- 2.2 mm Older age group Males: R-1.9 mm L- 1.8 mm Females: R- 1.8 mm L- 1.7 mm	-Diameter is more in males - Diameter is more in younger age group
Maaly et al. (2011) [31]	MRA	250	N.A.	Males- 1.18±0.07 mm Females- 1.12±0.07 mm Younger age group- 1.18±0.07 mm	-
Aggarwal et al. (2016)[29]	MRA	50	N.A.	Males: R- 2.42±0.85 mm L- 2.7±0.95 mm Females: R- 2.2±0.55 mm L- 2.3±0.82 mm	-Diameter of males is more
Vohra et al. (2006) [27]	Cadaveric MRA	30	N.A.	Cadaveric: 0.5Å-2.5 mm MRA: 1.0 mm-2.5 mm	-

Discussion

The length and diameter of ACA are very crucial as they can be used to investigate pathological conditions. There is a considerable individual variation in the pattern and caliber of vessels that make up the circulus arteriosus. Although a complete circular channel almost always exists anatomically, one vessel is usually sufficiently narrowed to reduce its role as a collateral route and the circle is rarely functionally complete in individuals [32].

Arteries of the left side (ACA) appear to be nearly the same as those of the right one, as the increase of perimeter of the arterial polygon is achieved by means of a harmonious increase of all its sides.

In our review of 21 articles; we observed that the range of length and diameter of ACA were 8.1 mm-21 mm and 0.5 Å-3.4 mm, respectively. In the majority of the studies the length and diameter of ACA were more in the younger age group (>40 years); and the length of ACA was more in females whereas the diameter of ACA was more in males.

Sex-Wise Distribution of Morphometry of ACA

Males’ head measures more than females’. Brain weight is more in males [33]. This might be the reason behind ACA diameter being more in males in most of the included studies. According to Poiseuille’s equation, blood flow is directly proportional to the diameter and length of the blood vessel [34]. Hence if the diameter of ACA is more in males, the length of ACA should be less compared to females. This is the reason behind the result of the study with the length of ACA being more in females. But this is not statistically correlated with the potential flow of the arterial polygon.

Age-Wise Distribution of Morphometry of ACA

Sympathomimetic activity increases with age leading to vasoconstriction; which may be the reason for the decrease of diameter and length with age. This effect may be overpowering the compensative widening of centripetal vessels in elderly people. Age-related differences in ACA had been explained by a compensative widening of the centripetal vessels in elderly people [35]. This may occur as a result of:

· Decrease in stroke volume of the heart.

· Weakening of the elasticity of arterial walls.

· Presence of arteriosclerotic changes.

These factors are also responsible for the decrease in the velocity of blood flow in vessels in elderly people [36].

Clinical implications

The ACA has a high incidence of hypoplasia or aplasia; and hence is more prone to ischemic complications [37]. Hypoplasia of ACA can lead to further narrowing of the ACoA too. ACA anomalies are more commonly found in ruptures as ACoA aneurysms. In patients with unilateral ICA blockage, blood flow is through anterior CW. This points out the fact that ACA is the favored path for supply in such cases [38]. Morphometric parameters of ACA can also be used to plan and design devices such as angiographic microcatheters and guides which are used in endovascular procedures. Thus, of clinical and surgical significance for neurosurgeons and radiologists. Emotionlessness may be caused due to ACA blockage. Moreover, control of pelvic muscles and perineal sensation may also be lost in ACA blockage. ACA blockage will also affect micturition [24].

Limitations of the study

This study required a great range of evidence from diverse databases with peer-reviewed material and gaining entry in various database was problematic. Moreover, all the articles included were not pertaining to all the factors (like length, diameter, age, sex).

## Conclusions

After observing the data on morphometry of ACA of all the studies, we conclude that there is a wide range of length and diameter of ACA, so it is difficult to find specific values for length and/or diameter. These data will be applicable for better construction and decipherment of angiographic images. The maximum number (31.8%) of the studies has a length in the range of 13.5 mm to 14.5 mm. As far as the diameter is concerned, the maximum number (27.3%) of the studies observed diameter of ACA to be nearly 2.5 mm. These findings of the basic knowledge of ACA are essential for interventional radiologists and neurosurgeons for planning the management of the patient.
